# A Collaborative Dictionary Learning Model for Nasopharyngeal Carcinoma Segmentation on Multimodalities MR Sequences

**DOI:** 10.1155/2020/7562140

**Published:** 2020-08-28

**Authors:** Haiyan Wang, Guoqiang Han, Haojiang Li, Guihua Tao, Enhong Zhuo, Lizhi Liu, Hongmin Cai, Yangming Ou

**Affiliations:** ^1^School of Computer Science and Engineering, South China University of Technology, 510000, China; ^2^Department of Radiology, State Key Laboratory of Oncology in South China, Collaborative Innovation Center for Cancer Medicine, Sun Yat-sen University Cancer Center, Guangzhou, 510060 Guangdong, China; ^3^Boston Children's Hospital, Harvard Medical School, Boston, MA 02115, USA

## Abstract

Nasopharyngeal carcinoma (NPC) is the most common malignant tumor of the nasopharynx. The delicate nature of the nasopharyngeal structures means that noninvasive magnetic resonance imaging (MRI) is the preferred diagnostic technique for NPC. However, NPC is a typically infiltrative tumor, usually with a small volume, and thus, it remains challenging to discriminate it from tightly connected surrounding tissues. To address this issue, this study proposes a voxel-wise discriminate method for locating and segmenting NPC from normal tissues in MRI sequences. The located NPC is refined to obtain its accurate segmentation results by an original multiviewed collaborative dictionary classification (CODL) model. The proposed CODL reconstructs a latent intact space and equips it with discriminative power for the collective multiview analysis task. Experiments on synthetic data demonstrate that CODL is capable of finding a discriminative space for multiview orthogonal data. We then evaluated the method on real NPC. Experimental results show that CODL could accurately discriminate and localize NPCs of different volumes. This method achieved superior performances in segmenting NPC compared with benchmark methods. Robust segmentation results show that CODL can effectively assist clinicians in locating NPC.

## 1. Introduction

Nasopharyngeal carcinoma (NPC) is an enigmatic malignancy with marked racial and geographical differences, being particularly prevalent in southern China, Southeast Asia, and northern Africa [1, 2]. Although advances in therapeutic techniques have contributed to improve clinical outcomes for patients with NPC, the mortality rate remains high. Early detection and accurate tumor localization of NPC are vital for surgical planning. Magnetic resonance imaging (MRI) is the first choice in primary tumor delineatio and a presurgical tool for localization and evaluation of the tumor entity [3–5]. In practice, the patient is usually scanned by T1-weighted (T1-w) or T2-weighted (T2-w) MR imaging. The T2-weighted (T2-w) imaging provides better fine structural information on soft tissues than by T1-w imaging. A contrast-enhanced T1-weighted (CET1-w) imaging is sometimes operated to provide direct evidence on tumor occurrence. Currently, identification and comprehensive assessment of the carcinoma entity NPC remain a great challenge. The infiltrative and migratory characteristics of NPC make it difficult to be discriminated from surrounding tissues.

To achieve automatic (or semiautomatic) segmentation of the NPC, traditional image processing has been used to fulfill the task. For example, [6] proposed a semiautomatic workflow, including masking, thresholding, and seed growing, to segment NPC from both T2-w and CET1-w from 7 patients to help radiation therapy. [7] proposed an automatic NPC segmentation method based on region growing and clustering and used neural networks to classify suspicious regions. [8] proposed to use a genetic algorithm for selecting the informative features and the support vector machine for classifying NPC. With the great success of deep learning models in computer vision, [9] proposed to use deep convolutional neural networks and graph cut on T1-w images from 30 NPC patients. [10] tested a deep deconvolutional neural network, composing of an encoder network and a decoder network, on CT images from 230 patients. [11] reported an automatic NPC segmentation method based on the convolutional neural network (CNN) architecture with dynamic contrast-enhanced MRI. [12] used fully convolutional networks with auxiliary paths to achieve automatic segmentation of NPC on PET-CT images. [13] used a modified U-Net model to automatically segment NPC on CT images from 502 patients. [14] proposed an automated method based on CNN for NPC segmentation on dual-sequence MRI (i.e., T1-w and T2-w) from 44 patients. Furthermore, the tumor volume varies greatly and many of them are small. Such sample characteristics raise a large difficulty in constructing representative learning models using deep networks.

**Figure 1:**
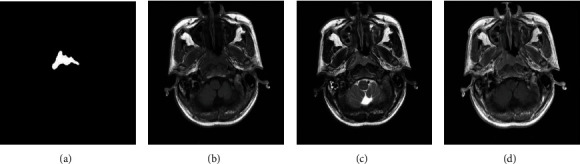
Example MR slices with three sequences. From left to right: (a) ground truth, (b) T1-w, (c) T2-w, and (d) CET1-w.

Recently, multiview learning models have been developed to analyze images from various imaging modalities or views. Fruitful advances have been made in reconstruction, face recognition, human motion recognition, and other object recognition issues [15–17]. In the current study, each patient underwent MRI by three sequences (i.e., T1-w, T2-w, and CET1-w) to enjoy the merits of different imaging characteristics (see Figure 1). The study is aimed at achieving the identification and segmentation of the NPC with high accuracy. Different views usually provide supplemental information. The problem of NPC segmentation can be formulated as a voxel-wise dictionary learning problem with three different views.

**Figure 2:**
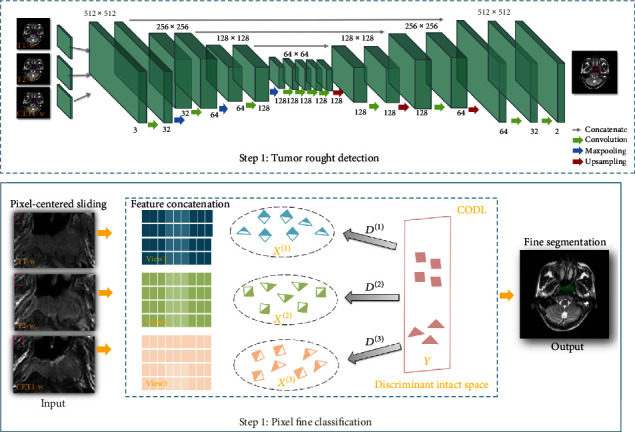
The workflow of location and segmentation of NPC. It consists of two steps, rough location by FCN and pixel-wise fine classification by CODL.

However, existing multiview learning methods cannot be tailored directly to be applied in NPC localization and segmentation. From the methodological aspect, most NPCs only occupy a small area in the entire slice. Such imbalance also results in a high false positive rate in applying learning models directly. To solve this difficulty, we preprocessed the data, that is, using a specially designed deep learning model with a fully convolutional network (FCN) structure to roughly locate the suspicious tumor area. In light of the advantages of multiview subspace learning, we propose to use a multiview learning collaborative dictionary model, which we call CODL, to further refine the detailed structure of NPC. The flowchart of NPC segmentation is illustrated in Figure 2.

The major contributions of our work are as follows:

1. An original collaborative dictionary model for multiview learning (CODL) is proposed to achieve fine segmentation. The CODL integrates cooperative information from multiple views to find latent intact space for the data and renders the latent space discriminative. The latent space is constructed by collaborative dictionary learning incorporating membership to possess discriminative power. Our approach takes into account the label of the samples to latent intact space. This gives a consistent indicator matrix discriminative capability
2. The numerical scheme involved in solving the CODL is provided. It treated the proposed unified framework into solvable subproblems, each with an explicit solution and a fast computation
3. While using all three MR sequences (T1-w, T2-w, and CET1-w) achieved the highest accuracy, we show that, for patients having kidney diseases that prevent the use of contrast agent necessary in CET1-w imaging [18, 19], using T1-w and T2-w alone does not significantly undermine the segmentation accuracy. This highlights the sensitivity and stability of the proposed CODL algorithm and improves the applicability of the proposed framework

## 2. Literature Review

Sparse codes generated by dictionaries can be directly used as features to train classifiers for recognition [20]. This two-stage scheme has been extensively used in many existing methods [21–25], such method uses the discriminative nature of sparse representation to perform classification. However, generated sparse codes are often insufficiently discriminative in complex recognition tasks [26]. One alternative is to unify dictionary learning and classifier training in an optimization model [27]. However, most of the supervised dictionary methods only employ single-view information in the learning process, which will result in the data not having the optimal expressibility. Besides, the model will also depend on the peculiarities of training data.

A naive way of multiview learning is feature fusion [28]. However, consolidating each single view may be suboptimal if the different views belong to different sample spaces. To address the drawback, weighted combinations [29] have been proposed. Alternatively, recent advances are aimed at learning the multiview data via finding an intact space, such as the multiview intact space learning (MISL) [15] and the multiview discriminant analysis with view consistency (MvDA-VC) [16]. In such approaches, a latent subspace shared by multiple views is learned by assuming that the input views are generated from this latent subspace. For multiview intact space learning, however, class membership is seldom used to find the optimal latent subspace and has little power available to handle problems in supervised learning.

As the goal of this paper is to develop a multiview dictionary learning method for voxel-wise classification. We first give a brief review of the supervised dictionary and multiview subspace learning methods related to our work. In this paper, we propose a novel collaborative dictionary model for multiview learning, which also takes into account the label of the samples to latent intact space. The construction of the latent space is guided by the supervised dictionary learning within each individual view and equipped to have discriminative power.

## 3. Materials and Methods

### 3.1. Dataset

A total of 24 patients with nonmetastatic NPC at the Sun Yat-sen University Cancer Center (SYSUCC) were enrolled in this study. MRI was performed on a 3.0-T scanner (Discovery MR750; GE Healthcare, Milwaukee, WI, USA). The imaging parameters are as follows: axial T1-w imaging (FSE, TR = 540 ms, and TE = 11.8 ms), axial T2-w imaging (FSE, TR = 4000 ms, and TE = 99 ms), and axial CET1-w imaging (FSE, TR = 540 ms, and TE = 11.8 ms). The number of slices per patient was 16, 32, or 36. Not every layer of MR images has lesions. The interval between each layer of images is 5 mm, in which imaging has a high resolution of 0.43 mm × 0.43 mm. T1-w, T2-w, and CET1-w MR sequences were assessed for each patient. Regions of interest (ROI) were drawn by four experienced radiologists (>3 years of clinical experience) using semiautomatic methods. They were required to draw all discernable tumor regions cautiously along axial directions. Any disagreements were resolved through negotiating until full consent was derived by the four.

The purpose of this study is to develop a multiview dictionary learning method for voxel-wise classification. We first give a rigid quality control on the selection of slices. Following the principle of multiple modalities sequences alignment, in total, 90 slices covering 30 instances of distinct tumor sizes were selected for our experiment. Each instance has three MR sequences (i.e., T1-w, T2-w, and CET1-w) and well-aligned before feeding into models.

### 3.2. A Collaborative Dictionary Model for Multiview Classification (CODL)

In this paper, we proposed a collaborative multiview learning model to fuse multiple image modalities into a consolidated space. By integrating each single modality and exploiting its characteristics comprehensively, the information among different modalities is actively learned and reinterpreted in a latent space. The supervised membership is used to render the latent space being discriminative, and thus, the sample classification is finally conducted within the learned latent space.

#### 3.2.1. Formulation of Multiview Collaborative Classification Model

Mathematically, let *X*^(*v*)^ = [*x*_1_^(*v*)^, *x*_2_^(*v*)^, ⋯, *x*_*s*_^(*v*)^] ∈ ℝ^*n*×*s*^(*v* = 1, 2, ⋯, *m*) denote a dataset containing *s* samples from the  *v*th view, with each sample characterized by a *n*-dimensional vector. We want to consolidate the multiview data into a latent space, denoted by *Y* = [*y*_1_, *y*_2_, ⋯, *y*_*s*_] ∈ ℝ^*d*×*s*^, where *d* is the dimensionality of the latent space.

Let *D*^(*v*)^ ∈ ℝ^*n*×*d*^(*v* = 1, 2, ⋯, *m*) denote the dictionary learned in the  *v*th view. The label for the training samples is denoted by  *L*. Our aim is to learn an informative latent space from multiple modalities and then achieve accurate classification task within the latent space. To this end, we proposed the following model to achieve latent space learning and classification simultaneously.

(1) $$\begin{array}{c} \underset{Y,D^{\left(v\right)},\beta }{\text{argmin}}\overset{m}{\underset{v=1}{\sum }}\frac{1}{2}{\left\|X^{\left(v\right)}-D^{\left(v\right)}Y\right\|}_{2}^{2}+\frac{1}{2}\lambda_{1}{\left\|L-Y^{T}\beta \right\|}_{2}^{2}+\lambda_{2}{\left\|\beta \right\|}_{1}. \end{array}$$

The first term in Equation (1) controls data fidelity by minimizing the reconstruction errors in the latent space *Y* through the dictionary *D*^(*v*)^. The second term renders the latent space with discriminative power. The two terms work collaboratively to yield a sharable latent space for different views. The third term encourages the loading coefficient *β* to be sparse to achieve economic expression. Besides, it also helps to stabilize the optimization due to large freedom in the objective function. The hyperparameters *λ*_1_ and *λ*_2_ are aimed at penalizing the reconstruction error and sparsity.

Once we obtain the learned dictionaries *D*^(*v*)^(*v* = 1, 2, ⋯, *m*) and the latent space *Y*, we can map a query sample *q*_*i*_ ∈ ℝ^*n*^ to its representation $\hat{q}\in {\mathbb{R}}^{d}$ in the latent space. The latent representation $\hat{q}$ is estimated by minimizing the following energy function:

(2) $$\begin{array}{c} \underset{\hat{q}}{\text{argmin}}\overset{m}{\underset{v=1}{\sum }}\frac{1}{2}{\left\|q_{i}-D^{\left(v\right)}\hat{q}\right\|}_{2}^{2}. \end{array}$$

Finally, we can classify the sample $\hat{q}$ in the latent space *Y* using benchmark classification models, e.g., *k*-nearest neighbor.

The proposed CODL not only integrates complementary information in multiple views to find a latent intact space for the data but also renders the latent space discriminative.

#### 3.2.2. Numerical Scheme for Solving CODL

The objective function Equation (1) is convex with respect to *D*^(*v*)^ and *Y*. Therefore, we used a heuristic alternating direction method to solve it. By minimizing one variable while fixing the others, the alternating direction method iteratively updates each variable until convergence. The alternate minimization method enjoys an excellent characteristic. It can decompose a large complex problem into small-sized subproblems, thus enabling parallel solving to have a quick convergence. In particular to our problem, it decomposes Equation (1) into three subproblems with respect to the three variables *D*^(*v*)^, *Y*, and *β*.

Step 1 to update *D*^(*v*)^: by fixing *Y* and *β* and discarding irrelevant terms, the objective function Equation (1) could be simplified as

(3) $$\begin{array}{c} \underset{D^{\left(v\right)}}{\text{argmin}}\overset{m}{\underset{v=1}{\sum }}\frac{1}{2}{\left\|X^{\left(v\right)}-D^{\left(v\right)}Y\right\|}_{2}^{2}. \end{array}$$

It is convex and differentiable with respect to the variable *D*^(*v*)^. By setting the gradient to zero, one has an explicit solution:

(4) $$\begin{array}{c} D^{\left(v\right)}=X^{\left(v\right)}Y^{T}{\left(YY^{T}\right)}^{-1}. \end{array}$$

Step 2 to update *β*: by fixing the variables  *D*^(*v*)^ and *Y*, the objective function Equation (1) could be simplified as:

(5) $$\begin{array}{c} {\left.\underset{\beta }{\text{argmin}}\frac{1}{2}\lambda_{1}\left\|{\left.L-Y^{T}\beta \right\|}_{2}^{2}+\lambda_{2}\right\|\beta \right\|}_{1}. \end{array}$$

It resembles the classical least absolute shrinkage and selection operator (LASSO) problem. By using a proximal gradient, its solution could be obtained by the iterative soft-thresholding algorithm (ISTA) [30]:

(6) $$\begin{array}{c} \;\beta^{k}=\underset{\beta }{\text{argmin}}\frac{1}{2}{\left\|L-Y^{T}\beta \right\|}_{2}^{2}+{\left\|\beta \right\|}_{1}=S_{\left(\lambda_{1}/\lambda_{2}\right)t}\left(\beta^{\left(k-1\right)}+tY\left(L-Y^{T}\beta^{\left(k-1\right)}\right)\right), \end{array}$$

where  *t*  is the step size and *S*_*λt*_(*β*) is the soft-thresholding operator. One could further accelerate the ISTA to achieve fast convergence

(7) $$\begin{array}{c} \beta^{\left(k\right)}=S_{\left(\lambda_{1}/\lambda_{2}\right)t}\left(g+tY\left(L-Y^{T}g\right)\right),g=\beta^{\left(k-1\right)}+\frac{k-2}{k+1}\left(\beta^{\left(k-1\right)}-\beta^{\left(k-2\right)}\right). \end{array}$$

Step 3 to update *Y*: by fixing *D*^(*v*)^ and *β*, the objective function Equation (1) could be simplified as

(8) $$\begin{array}{c} \underset{Y}{\text{argmin}}\overset{m}{\underset{v=1}{\sum }}\frac{1}{2}{\left\|X^{\left(v\right)}-D^{\left(v\right)}Y\right\|}_{2}^{2}+\frac{1}{2}\lambda_{1}{\left\|L-Y^{T}\beta \right\|}_{2}^{2}. \end{array}$$

Setting the gradient with respect to *Y* to be zero, one has

(9) $$\begin{array}{c} Y={\left(\overset{m}{\underset{v=1}{\sum }}{\left(D^{\left(v\right)}\right)}^{T}D^{\left(v\right)}+\lambda_{1}\beta \beta^{T}\right)}^{-1}\left(\overset{m}{\underset{v=1}{\sum }}{\left(D^{\left(v\right)}\right)}^{T}X^{\left(v\right)}+\lambda_{1}\beta L^{T}\right). \end{array}$$

The above three schemes are iteratively updated until convergence.

In the testing phase, one needs to find the new representation $\hat{q}$ for query samples *q* through the dictionary *D*^(*v*)^ by solving Equation (2). It is a standard least square minimization problem with an explicit solution

(10) $$\begin{array}{c} \hat{q}={\left[\overset{m}{\underset{v=1}{\sum }}\left(D^{\left(v\right)}D^{\left(v\right)}\right)\right]}^{-1}\overset{m}{\underset{v=1}{\sum }}D^{\left(v\right)}q. \end{array}$$

The pseudocode for solving CODL is provided in Algorithm 1.

#### 3.2.3. Complexity Analysis

The computational time of solving the proposed model is mainly taken by updating the *D*^(*v*)^, *β*, and *Y*. As mentioned in Section 3.2.1, *D*^(*v*)^ ∈ ℝ^*n*×*d*^, *β* ∈ ℝ^*d*×1^, and *Y* ∈ ℝ^*d*×*s*^, where *n* is the dimensionality of the *v*th view, *d* is the dimensionality of the latent space, and *s* is the number of multiview objects. According to Algorithm 1, the main computational cost of CODL is incurred in the iterative calculations of *D*^(*v*)^, *β*, and *Y*. In each inner iteration, the computational cost of solving *D*^(*v*)^ by Equation (4) is *O*(*nsd* + *d*^2^*s* + *d*^3^ + *nd*^2^), the computational cost of solving *β* by Equation (6) is *O*(*d*^3^ + *d*^2^*s*), and the computational cost of solving *Y* via Equation (9) is *O*(*d*^2^*n* + *d*^2^*s* + *dns* + *d*^3^). Therefore, the total computational complexity is *O*(*dns* + *d*^2^*n* + *d*^2^*s* + *d*^3^).

## 4. Experiments and Results

We applied the proposed model on both a synthetic dataset and a real NPC dataset. For a fair comparison, each method was run on the synthetic data 10 times, and the averaged results were recorded. On a real NPC dataset, we tested the performance of each method using 10-fold cross-validation scheme. Classification accuracy was measured in terms of average accuracy across ten trials on different training and testing sets. Moreover, the parameters in each compared method are tuned to meet the best performance in the suggested range. For CODL, we empirically set the parameters, that is, *λ*_1_ = 0.01, *λ*_2_ = 0.7 for single view, *λ*_1_ = 1.0, *λ*_2_ = 0.2 for two views, and *λ*_1_ = 3.8, *λ*_2_ = 0.2 for three views, throughout all experiments. All of our experiments were performed on a desktop computer with a 4.20 GHz Intel(R) Core (TM)i7-7700K CPU, 16.0 GB of RAM, and MATLAB R2017a (×64).

### 4.1. Evaluation Metrics and Baseline Methods for Performance Comparisons

Six widely used metrics, including the sensitivity (SENS), the dice similarity coefficient (DICE), the area under the receiver operating characteristic curve (AUC), intersection over union (IoU), mean pixel accuracy (MPA), and Hausdorff distance (HD) were employed to measure the performances of each tested method. These qualitative metrics were defined as follows:

(11) $$\begin{array}{c} \text{SENS}=\frac{\text{TP}}{\text{TP}+\text{FN}},\\ \text{DICE}=\frac{2\text{TP}}{\text{TP}+\text{FN}+\text{TP}+\text{FP}},\\ \text{IoU}=\frac{\text{TP}}{\text{TP}+\text{FP}+\text{FN}},\\ \text{MPA}=\frac{\text{TPR}+\text{TNR}}{2}\;, \end{array}$$

where  TP, FP,  TN, FN, TPR, and TNR represented true positive, false positive, true negative, false negative, true positive rate, and true negative rate, respectively. We also plotted the receiver operating characteristic curve (ROC) for each method. The area under the ROC curve (AUC) was then estimated. For two point sets A  and *B*, the Hausdorff distance between these two sets is defined as follows:

(12) $$\begin{array}{c} \text{HD}\left(A,B\right)=\max\left(\text{hd}\left(A,B\right),\text{hd}\left(B,A\right)\right)\;, \end{array}$$

where $\text{hd}\left(A,B\right)=\underset{x\in X}{\max}\underset{y\in Y}{\;\min\;}{\left\|x-y\right\|}_{2}$, $\text{hd}\left(B,A\right)=\underset{y\in Y}{\max}\underset{x\in X}{\;\min\;}{\left\|x-y\right\|}_{2}$. For this study, we have used the Euclidean norm ‖*x* − *y*‖_2_.

Several benchmark methods are borrowed to serve as baseline methods for comparisons. They are widely used multiview methods and most relevant to our method.

- Support vector machine (SVM) [31]: we concatenate the features of all views and perform support vector machine classification
- Multiview intact space learning (MISL) [15]: it is aimed at integrating the encoded complementary information from different views into a latent intact space. It shows theoretically that combining multiple views can obtain abundant information for latent intact space learning
- Multiview discriminant analysis with view consistency (MvDA-VC) [16]: it seeks for a single discriminant common space for multiple views in a nonpairwise manner by jointly learning multiple view-specific linear transforms. MvDA-VC method has achieved good performance in addressing the problem of object recognition from multiple views
- Zhao et al. [12]: it uses fully convolutional networks with an auxiliary path to achieve automatic segmentation of NPC on dual-modality PET-CT images. The proposed method improves NPC segmentation by guiding the training of lower layers by auxiliary paths
- Li et al. [13]: it proposes a modified version of the U-Net, which performs well on NPC segmentation by modifying the downsampling layers and upsampling layers to have a similar learning ability and predict the same spatial resolution as the source image

### 4.2. Discriminative Capability Tests of CODL on Synthetic Data

We first constructed a synthetic data to test the discrimination power of the proposed methods. The synthetic data consisted of three classes, and they were separable within a three-dimensional space, but inseparable when projected orthogonally into two-dimensional (2D) plane (i.e., *X*-*Y* and *Y*-*Z* planes). The projected samples into each 2D plane were considered an observed individual view. The synthetic data contained 3000 samples from three classes, each following a multivariate normal distribution with mean values *μ*_1_ = (10 20 30), *μ*_2_ = (10 20 35), *μ*_3_ = (16 20 35), and covariances

(13) $$\begin{array}{c} \sum =\left(\begin{array}{ccc} 1 & 0 & 0\\ 0 & 1 & 0\\ 0 & 0 & 1 \end{array}\right), \end{array}$$

respectively.

**Figure 3:**
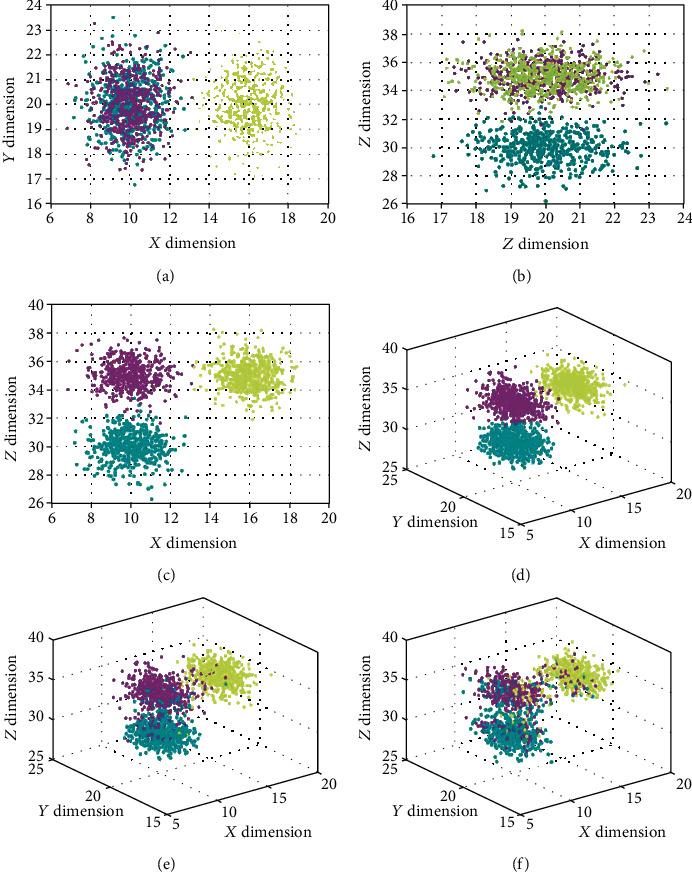
A toy example to demonstrate the discrimination power of the CODL. The data is collected from three views on (a) *X*-*Y* plane, (b) *Y*-*Z* plane, and (c) *X*-*Z* plane. The reconstructed results in *X*-*Y*-*Z* space by CODL on (d) the intact noiseless data, (e) the noisy data with std = 0.5, and (f) its std = 1 noisy counterpart are also shown. Different classes are highlighted in different colors.

To test the robustness of the model over noise contaminations, the synthetic data were corrupted by Gaussian white noises with a standard deviation of 0.25, 0.5, and 1, respectively. The synthetic data was shown in Figure 3. The first row was the three different views along different planes (i.e., *X*-*Y*, *Y*-*Z*, and *X*-*Z* planes), respectively. The corresponding classified results by the proposed CODL were shown in the second row of Figure 3. Classification performance was measured in terms of average accuracy across ten trials. The percentage of training sets and test sets in each trial is 1 : 1. The averaged results were recorded and summarized in Table 1.

Since the individual view cannot reveal the intrinsic structure of the data, one may note that the classification on each individual view may not obtain accurate results. When the synesthetic data was noise free, the classification by MvDA-VC obtained the highest accuracy by fusing *X*-*Y*, *Y*-*Z*, and *X*-*Z* views. However, when the noise level increased, its performances were inferior to the MISL and CODL. Throughout the experiments, the proposed CODL achieved the best performance uniformly. With the increasing noise level, the reduction of our method's classification performance was significantly lower than that of other methods. Even when the data was heavily contaminated by the noises (std = 1), the CODL remained superior performance with the highest accuracy of 74.9%.

### 4.3. Realistic Experiments on Nasopharyngeal Carcinoma Data

#### 4.3.1. Image Preprocessing

Most of the NPCs have a small volume and thus are very difficult to discriminate from its large surrounding. Such imbalance also results in a large false positive rate in applying learning models directly. To solve these difficulties, we firstly designed a fully convolutional network (FCN) to locate a rectangular box bounding the suspicious tumor. The network contains standard layers, including convolution, maximum pooling, and upsampling [32]. Our network used a jump structure to exploit deep and shallow semantic information. It also used multiscale convolution kernels to obtain a comprehensive global structure. The network was trained to predict a rectangular bounding box for the NPC.

**Figure 4:**
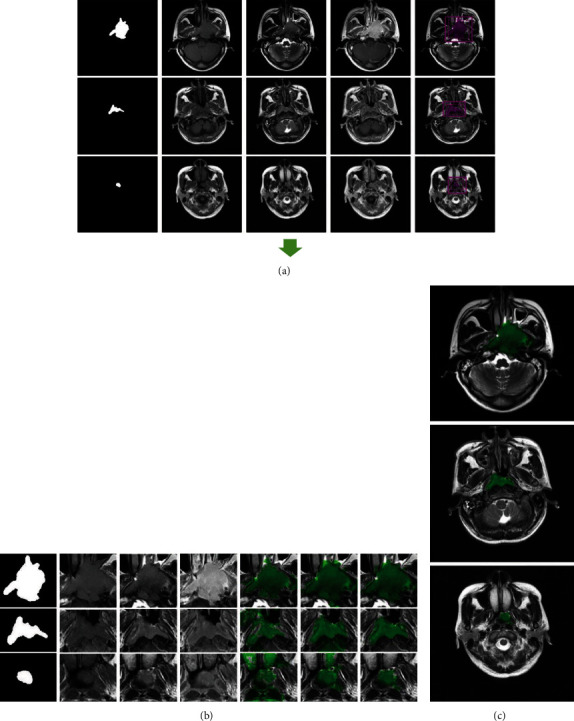
NPC segmentation results on three typical examples. (a) Rough location results with bounding boxes identified by FCN, highlighted in red dots. The extended areas used for fine classification were indicated by solid red lines. (b) Fine segmentation results with fusing T1-w, T2-w, and CET1-w MR sequences. The last three columns are the tumor regions located by MISL, MvDA-VC, and CODL, respectively. (c) Results of our method on the whole slice in case of a combination of three modalities.

The detailed architecture of the FCN network for NPC location is summarized in Table 2.  Figure 4(a) showed the MR slices with bounding boxes identified by FCN, highlighted in red dots. We selected an outer area by extending the located bounding box by fifteen pixels outward to ensure that it sufficiently covers the tumor region.

#### 4.3.2. Radiomics Feature Extraction and Classification

In the bounding box, each voxel is classified into a binary label of tumor vs. normal. The features for each pixel were estimated within a sliding window of 11 × 11 centered itself. A total of 192 radiomics features (i.e., 32 Gabor, 5 Momentum, 154 GLCM, and 1 Pixel) were extracted for each sliding window. See section S1 in the Supplementary Material for more information on radiomics feature. If the border size is 103 × 78, it resulted in a sample matrix with 8034 samples and 192 features. The methods for extracting features from T1-w, T2-w, and CET1-w sequences are the same. We use *z*-score for standardization. Finally, we use an adaptive median filter function to perform a simple postprocessing on the entire slice to retain the largest connected area.

We tested the performance of CODL using a 10-fold cross-validation scheme. The percentage of training sets and test sets per fold cross-validation is 9 : 1. A total of 30 instances (training cohort: 27, testing cohort: 3) were enrolled in the voxel classification analysis. Classification accuracy was measured in terms of average accuracy across ten trials on different training and testing sets.

#### 4.3.3. Experimental Results

**Figure 5:**
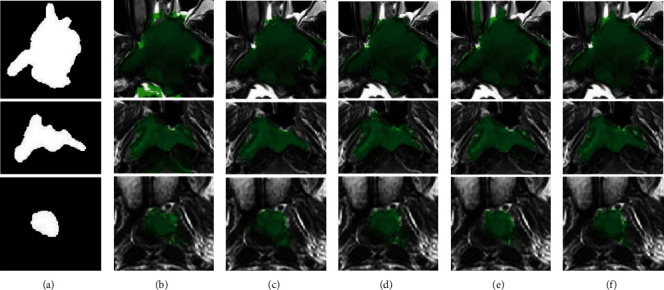
Typical segmentation results of three instances using CODL. (a) Ground truth. From second to last column: the tumor regions identified by CODL on modality T1-w (b), T2-w (c), CET1-w (d), both T1-w and T2-w (e), and T1-w, T2-w, and CET1-w (f), respectively.

Figure 5 visualizes NPC segmentation results on three typical instances, having large, medium, and small size tumors, respectively. Each row stands for segmentation results for one instance of MR sequences. From Figure 5, one would find that the segmentation results of CODL with fusing T1-w, T2-w, and CET1-w MR sequences obtained a highly accurate segmentation.

Figure 4 shows the overall segmentation process. As is illustrated in Figure 4(a), we select the outer area by expanding the positioned bounding box 15 pixels outward. The extended areas used for fine classification were indicated by solid red lines.  Figure 4(b) shows pixel-wise fine classification results using MISL, MvDA-VC, and CODL. One may observe that CODL obtained the highest accuracy. Our method performed stably in identifying tumors of different volumes. Specifically, Figure 4(c) showed the identified tumors in the whole slices. One may observe that the proposed method identifies the tumor successfully with its boundary almost perfectly overlapped with the actual one.

We report the detailed numerical results on cropped NPC dataset in Table 3.

In the first section in Table 3, we firstly tested the classification performance on each individual image modalities. CODL performed uniformly better than SVM. The superior performance of CODL is consistent with the synthetic data. Moreover, the CET1-w provides a more accurate classification than T2-w or T1-w. The AUCs by CODL were 0.868 ± 0.050, 0.860 ± 0.090, and 0.847 ± 0.055 on CET1-w, T2-w, and T1-w, respectively.

Considering that some NPC patients do not get CET1-w scans due to kidney diseases, we used two modalities T1-w and T2-w to rerun the experiments. The results were summarized in the second section in Table 3. Overall, the accuracy has increased, which is higher than using any single MR modality. CODL with the fusion of T1-w and T2-w modalities scored the highest accuracy.

Finally, we used three MR modalities. One may observe that CODL achieved superior performances in classifying the nasopharyngeal carcinoma. The DICE, AUC, IoU, and MPA for CODL were uniformly larger than those by the other methods. Incorporating the imaging of CET1-w achieved minor improvement (0.889 ± 0.054) than without it (0.886 ± 0.055) by CODL. It implies that the CODL could exploit fully discriminative information in the modality of T1-w and T2-w, such that the loss of accuracy after dropping CET1-w is only mild.

**Figure 6:**
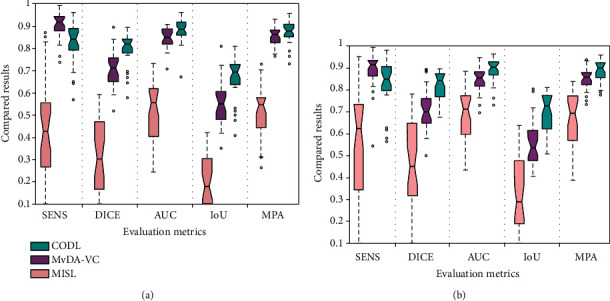
Quantitative results of MISL, MvDA-VC, and CODL on multiple sequences of (a) T1-w and T2-w and (b) T1-w, T2-w, and CET1-w.

Quantitative results of each method were shown by box plots in Figure 6. In terms of DICE, AUC, IoU, MPA, and HD, the performance of CODL is superior to the other methods. Another noticeable characteristic of the CODL lies in its robustness. One would find that the variances by the different metrics are dramatically smaller than by other methods. Such high robustness coincides with the experiments on synthetic data.

We report the detailed numerical results on whole MR slices in Table 4.

In the first section in Table 4, we firstly tested the segmentation performance on two modalities T1-w and T2-w. One may observe that our approach achieved superior performances in NPC segmentation.

Finally, we used three modalities (i.e., T1-w, T2-w, and CET1-w) to rerun the experiments. The results were summarized in the second section in Table 4. The SENS, DICE, AUC, IoU, and MPA for our approach were uniformly larger than other methods. There were good overlaps in DICE and HD values for our method between segmented contours and ROIs drawn by radiologists. By checking the results, one can find that the variances by the six metrics are dramatically smaller than by other methods.

**Figure 7:**
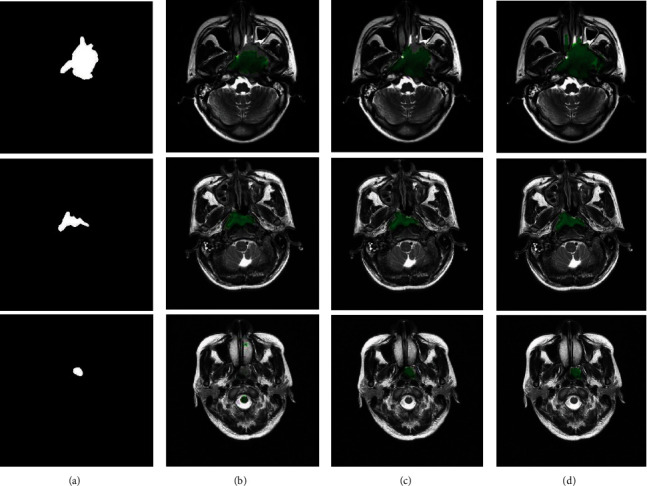
NPC segmentation results by fusing T1-w and T2-w modalities on the whole slices. (a) Ground truth. From second to last column: the tumor regions located by (b) Zhao et al. [12], (c) Li et al. [13], and (d) our approach, respectively.

Figure 7 shows NPC segmentation results in case of fusing two modalities (i.e., T1-w and T2-w). From Figure 7, one may observe that the proposed method identifies the tumor successfully with its boundary almost perfect overlapped with the ground truth drawn by radiologist. Our approach achieved superior performances in segmenting NPC compared with other methods.

**Figure 8:**
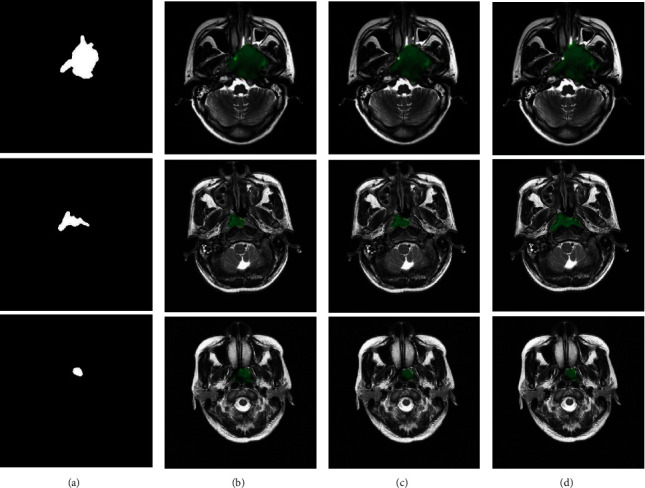
NPC segmentation results by fusing T1-w, T2-w, and CET1-w modalities on the whole slices. (a) Ground truth. From second to last column: the tumor regions located by (b) Zhao et al. [12], (c) Li et al. [13], and (d) our approach, respectively.

Figure 8 visualizes NPC segmentation results in case of fusing three modalities (i.e., T1-w, T2-w, and CET1-w). From Figure 8, one would find that the segmentation results of our approach obtained a highly segmenting performance. It can be seen that our approach could help make their wanting segmentation better.

## 5. Discussion

In our model, there are two regularization parameters (i.e.,  *λ*_1_ and *λ*_2_) balancing the effect of approximation error and sparse term. In the following, we study the influence of parameters *λ*_1_, *λ*_2_ on the NPC dataset in terms of SENS, DICE, AUC, IoU, and MPA by setting them to different values, e.g., [1, 2, ⋯, 10]. We vary a parameter at a time while keeping others fixed. Due to the limitation of space, we only show the results of a combination of two (i.e., T1-w and T2-w) and three modalities (i.e., T1-w, T2-w, and CET1-w).

**Figure 9:**
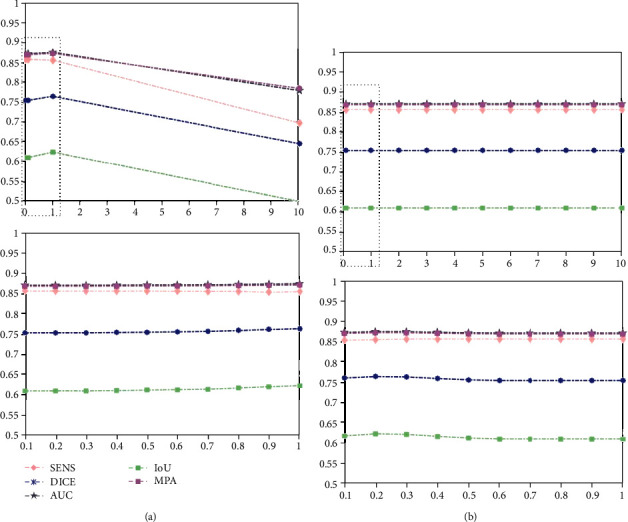
Performance of our model on NPC dataset with different parameter settings by fusing T1-w and T2-w modalities: (a) hyperparameter *λ*_1_ and (b) hyperparameter *λ*_2_.

**Figure 10:**
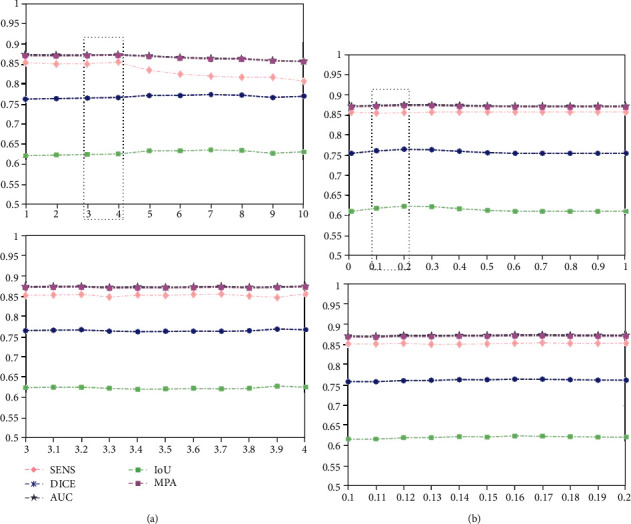
Performance of our model on the NPC dataset with different parameter settings by fusing T1-w, T2-w, and CET1-w modalities: (a) hyperparameter *λ*_1_ and (b) hyperparameter *λ*_2_.

From Figure 9, we can see that our method is relatively insensitive to its parameters as long as the parameters are in a suitable range. Moreover, we find that our method performs well when parameter  *λ*_1_ ∈ (0.1,1.0), *λ*_2_ ∈ (0.1,1.0). Thus, we select  *λ*_1_ = 1.0, *λ*_2_ = 0.2 in our experiment. Similarly, from Figure 10, we find that our method performs well when parameter *λ*_1_ ∈ (3.0,4.0), *λ*_2_ ∈ (0.1,0.2). Consequently, we choose *λ*_1_ = 3.8, *λ*_2_ = 0.2 for experiments.

## 6. Conclusions

In this study, we have proposed a voxel-wise classification method for locating and segmenting NPC from normal tissues. Specifically, each voxel is classified into a binary label of tumor vs. normal. The located NPC is refined to obtain its accurate segmentation by an original multiview collaborative dictionary classification model. The proposed CODL integrates complementary information from multiple views and collaboratively constructs a discriminative latent intact space through rendering with supervised membership. Experimental results show that CODL could accurately discriminate NPCs and effectively assist clinicians in locating NPC.

## Figures and Tables

**Algorithm 1:**
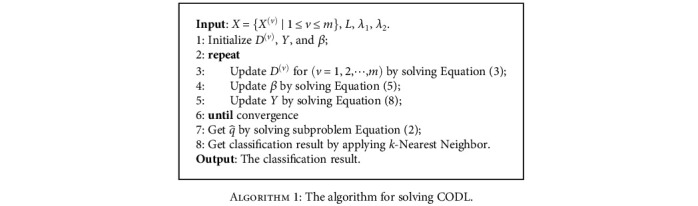
The algorithm for solving CODL.

**Table 1:** The performance of different methods on noisy synthetic datasets (mean ± standard deviation).

Method	Noise free	std = 0.25	std = 0.5	std = 1
SVM (V1)	0.664 ± 0.007	0.647 ± 0.008	0.614 ± 0.009	0.527 ± 0.013
SVM (V2)	0.606 ± 0.008	0.563 ± 0.009	0.561 ± 0.008	0.462 ± 0.034
SVM (V3)	0.938 ± 0.016	0.932 ± 0.016	0.843 ± 0.018	0.671 ± 0.021
CODL (V1)	0.668 ± 0.011	0.659 ± 0.007	0.621 ± 0.010	0.523 ± 0.014
CODL (V2)	0.664 ± 0.011	0.648 ± 0.013	0.591 ± 0.011	0.496 ± 0.012
CODL (V3)	0.994 ± 0.002	0.969 ± 0.005	0.874 ± 0.008	0.679 ± 0.014
SVM (FeaConcat)	0.946 ± 0.007	0.912 ± 0.007	0.843 ± 0.021	0.735 ± 0.042
MISL (fusion)	0.984 ± 0.004	0.966 ± 0.006	0.908 ± 0.011	0.733 ± 0.013
MvDA-VC (fusion)	0.995 ± 0.002	0.968 ± 0.005	0.876 ± 0.010	0.675 ± 0.011
CODL (fusion)	0.987 ± 0.002	0.971 ± 0.004	0.911 ± 0.006	0.749 ± 0.011

^∗^The V1, V2, and V3 denote *X*-*Y*, *Y*-*Z*, and *X*-*Z* views, respectively. ^∗^FeaConcat means that we concatenate features of all views to generate a combined feature. ^∗^Fusion means that we construct a multiview latent intact space learning by fusing all individual views. ^∗^Classification performance is measured in terms of average accuracy.

**Table 2:** Architecture of the FCN network for tumor localization.

	Type	Input size	Output size	Filter size	Stride	# filters
Layer 1	Conv.	512 × 512 × 3	512 × 512 × 32	3 × 3	1 × 1	32
Layer 2	Max-pool.	512 × 512 × 32	256 × 256 × 32	2 × 2	2 × 2	—
Layer 3	Conv.	256 × 256 × 32	256 × 256 × 64	5 × 5	1 × 1	64
Layer 4	Max-pool.	256 × 256 × 64	128 × 128 × 64	2 × 2	2 × 2	—
Layer 5	Conv.	128 × 128 × 64	128 × 128 × 128	7 × 7	1 × 1	128
Layer 6	Max-pool.	128 × 128 × 128	64 × 64 × 128	2 × 2	2 × 2	—
Layer 7	Conv.	64 × 64 × 128	64 × 64 × 128	3 × 3	1 × 1	128
Layer 8	Conv.	64 × 64 × 128	64 × 64 × 128	3 × 3	1 × 1	128
Layer 9	Conv.	64 × 64 × 128	64 × 64 × 128	3 × 3	1 × 1	128
Layer 10	Conv.	64 × 64 × 128	64 × 64 × 128	3 × 3	1 × 1	128
Layer 11	Upsampling	64 × 64 × 128	128 × 128 × 128	2 × 2	2 × 2	—
Layer 12	Conv.	128 × 128 × 128	128 × 128 × 128	7 × 7	1 × 1	128
Layer 13	Upsampling	128 × 128 × 128	256 × 256 × 128	2 × 2	2 × 2	—
Layer 14	Conv.	256 × 256 × 128	256 × 256 × 64	5 × 5	1 × 1	64
Layer 15	Upsampling	256 × 256 × 64	512 × 512 × 64	2 × 2	2 × 2	—
Layer 16	Conv.	512 × 512 × 64	512 × 512 × 32	3 × 3	1 × 1	32
Layer 17	Conv.	512 × 512 × 32	512 × 512 × 2	1 × 1	1 × 1	2

^∗^The convolutional layer is denoted by Conv., and the max pooling by max-pool.

**Table 3:** Metric results (mean ± standard deviation) of different methods on the cropped NPC dataset.

Method	SENS	DICE	AUC	IoU	MPA	HD
SVM (T1-w)	0.570 ± 0.292	0.558 ± 0.237	0.729 ± 0.107	0.419 ± 0.199	0.728 ± 0.113	28.566 ± 13.664
SVM (T2-w)	0.609 ± 0.269	0.652 ± 0.229	0.790 ± 0.144	0.518 ± 0.216	0.780 ± 0.134	23.212 ± 12.273
SVM (CET1-w)	0.733 ± 0.158	0.731 ± 0.093	0.827 ± 0.078	0.584 ± 0.112	0.829 ± 0.075	20.979 ± 8.505
CODL (T1-w)	0.832 ± 0.118	0.733 ± 0.088	0.847 ± 0.055	0.586 ± 0.110	0.847 ± 0.054	23.241 ± 9.168
CODL (T2-w)	0.812 ± 0.138	0.745 ± 0.119	0.860 ± 0.090	0.607 ± 0.145	0.854 ± 0.077	23.057 ± 10.456
CODL (CET1-w**)**	0.828 ± 0.102	0.767 ± 0.074	0.868 ± 0.050	0.627 ± 0.094	0.864 ± 0.047	22.827 ± 10.103
SVM (FeaConcat2)	0.377 ± 0.182	0.505 ± 0.204	0.692 ± 0.087	0.360 ± 0.168	0.682 ± 0.091	24.470 ± 13.020
MISL (Fusion2)	0.412 ± 0.254	0.310 ± 0.172	0.531 ± 0.136	0.195 ± 0.121	0.530 ± 0.115	38.052 ± 8.738
MvDA-VC (Fusion2)	0.901 ± 0.072	0.718 ± 0.090	0.853 ± 0.046	0.567 ± 0.111	0.858 ± 0.043	24.807 ± 7.010
CODL (Fusion2)	0.827 ± 0.094	0.808 ± 0.075	0.886 ± 0.055	0.683 ± 0.099	0.877 ± 0.052	16.618 ± 9.524
SVM (FeaConcat3)	0.211 ± 0.139	0.327 ± 0.194	0.611 ± 0.086	0.211 ± 0.139	0.606 ± 0.070	31.288 ± 16.200
MISL (Fusion3)	0.530 ± 0.262	0.452 ± 0.219	0.680 ± 0.123	0.317 ± 0.185	0.667 ± 0.125	32.490 ± 9.290
MvDA-VC (Fusion3)	0.893 ± 0.086	0.713 ± 0.094	0.846 ± 0.055	0.562 ± 0.116	0.853 ± 0.050	24.918 ± 7.243
CODL (Fusion3)	0.836 ± 0.111	0.820 ± 0.062	0.889 ± 0.054	0.699 ± 0.087	0.885 ± 0.049	16.683 ± 9.447

^∗^FeaConcat2 and Fusion2 denote concatenating and fusing T1-w and T2-w, respectively. ^∗^FeaConcat3 and Fusion3 denote concatenating and fusing T1-w, T2-w, and CET1-w, respectively.

**Table 4:** Metric results (mean ± standard deviation) of different methods on the whole slices.

Method	SENS	DICE	AUC	IoU	MPA	HD
Zhao et al. [12] (Fusion2)	0.723 ± 0.242	0.662 ± 0.160	0.814 ± 0.121	0.511 ± 0.149	0.858 ± 0.119	31.365 ± 19.268
Li et al. [13] (Fusion2)	0.469 ± 0.338	0.523 ± 0.300	0.723 ± 0.179	0.407 ± 0.274	0.734 ± 0.168	38.769 ± 28.383
Ours (Fusion2)	0.823 ± 0.096	0.804 ± 0.077	0.908 ± 0.048	0.678 ± 0.100	0.910 ± 0.048	16.918 ± 9.553
Zhao et al. [12] (Fusion3)	0.713 ± 0.223	0.664 ± 0.165	0.806 ± 0.125	0.518 ± 0.178	0.854 ± 0.110	30.388 ± 11.953
Li et al. [13] (Fusion3)	0.689 ± 0.237	0.741 ± 0.197	0.826 ± 0.128	0.618 ± 0.195	0.844 ± 0.118	21.928 ± 11.037
Ours (Fusion3)	0.828 ± 0.109	0.813 ± 0.066	0.910 ± 0.060	0.690 ± 0.090	0.913 ± 0.054	16.895 ± 9.624

^∗^Fusion2 denote fusing T1-w and T2-w. ^∗^Fusion3 denote fusing T1-w, T2-w, and CET1-w.

## Data Availability

The NPC image data used to support the findings of this study have not been made available for the private protection of patient information.

## Supplementary Materials

Supplementary Materials The details of the 192 radiomics features that used in this study.
