# Supplementary material for: A Collaborative Dictionary Learning Model for Nasopharyngeal Carcinoma Segmentation on Multimodalities MR Sequences
Source: Comput Math Methods Med. 2020 Aug 28;2020:7562140. doi: 10.1155/2020/7562140 (PMC7474760; doi:10.1155/2020/7562140)
Supplement: Supplementary Materials — The details of the 192 radiomics features that used in this study. [file 7562140.f1.docx]

# Supplementary Materials

# S1. Radiomics feature extraction

A total of 192 radiomics features (i.e., 32 Gabor, 5 Momentum, 154 GLCM, and 1 Pixel) were extracted for each sliding window. The methods for extracting features from T1-w, T2-w, and CET1-w sequences are the same. The detailed features are summarized as follows:

**1. Pixel feature**

We consider the pixel value of the central point of the sliding window as a feature of the image. A total of 1 pixel feature was extracted for each sliding window.

**2. Momentum features**

We use $n$ central moment features of the image with order from 1 to 5. A total of 1*5=5 momentum features were extracted for each sliding window.

**3. Statistics-based texture features**

We divided [-2^15, 2^15] into 16 levels and calculated 7 gray level co-occurrence matrices (GLCM) with offsets of [-3, -1; -1, 0; 0, 1; 0, 3; 1, -1; 1, 3; 2, -2] , and with angles of 0, 45, 90 and 135. For each GLCM, 22 statistical features were extracted, including energy, entropy, dissimilarity, contrast, inverse difference, correlation, homogeneity, autocorrelation, cluster shade, cluster prominence, maximum probability, sum of squares, sum average, sum variance, sum entropy, difference variance, difference entropy, two kind of information measures of correlation, maximal correlation coefficient, inverse difference normalized and inverse difference moment normalized. A total of 7*22=154 GLCM-related features were extracted from each sliding window.

**4. Gabor features**

We filtered each ROI sliding window using 32 Gabor filters with wavelength of 2.83, 5.66, 11.31, 22.63 and eight orientations to obtain 32 filtered images. A total of 4*8=32 Gabor features were extracted for each sliding window.

**After all the features were extracted, z-score normalization was applied to reduce feature biases among samples.**
